# Negotiating motherhood and authority: the experience of non-migrant wives in parenting their adolescent children from Filipino transnational families

**DOI:** 10.3389/fsoc.2025.1656817

**Published:** 2025-08-13

**Authors:** Peachy Domingo

**Affiliations:** Department of Social Sciences, College of Arts and Social Sciences, Central Luzon State University, Muñoz, Philippines

**Keywords:** Filipino mothering practices, Filipino transnational families, gender dynamics, Filipino adolescents, home-making, mother-adolescent relationship, overseas Filipino families

## Abstract

Transnational Filipino families arise from temporary labor migration, often with one parent abroad. This reshapes family life, impacting parent-child relationship and gender dynamics, and caregiving for children left-behind. However, non-migrant mothers’ crucial role in sustaining life and providing care is often overlooked in studies. This study examines how non-migrant wives in Filipino transnational families maintain and negotiate their authority in raising their adolescent children. This study utilized qualitative descriptive approach in conducting interviews with 20 Filipino mothers from transnational families in rural Philippines. Findings revealed that non-migrant mothers enforce their authority through various strategies such as the adoption of authoritarian parenting style, leveraging the authority of the Overseas Filipino Worker (OFW) husbands, chore delegation among adolescent children, and engage in negotiations regarding both their authority and their children’s autonomy. In examining Filipino transnational families, this study offers a novel perspective on maternal authority, situating its enforcement within the context of mothers’ significant and often equal financial contributions with their OFW husbands. This paper argues that maternal authority is profoundly shaped by the good motherhood ideology, where mothers actively promote children’s positive behavior and values as a testament to their own efficacy despite the absence of their OFW husbands. The unique contribution of this study lies in demonstrating how mothers primarily achieve this through consistent nagging and reprimanding their adolescent children. A mother’s financial contribution, relative to her husband’s remittances, significantly affects the effectiveness of her maternal control strategies and her perceived authority over their children. Furthermore, when their direct authority is insufficient, mothers strategically leverage the traditional disciplinarian role of their OFW husbands, highlighting a critical, yet under-explored, reciprocal dynamic: non-migrant wives reinforce the OFW father’s authority, while the OFW husband’s involvement simultaneously empowers the mother’s disciplinary actions. This intricate interplay of roles and financial contributions distinguishes our understanding of parental authority in transnational family structures.

## 1 Introduction

Transnational families emerge from temporary labor migration of a family member, leading to the reorganization of family life as members navigate relationships and new roles across distances (Jaji, 2022; König et al., 2024). The emergence of transnational families is largely driven by profound global disparities in income distribution and the availability of viable employment which reproduce new modes of transnational division of labor (Celero, 2022). The creation of transnational families in the Philippines is often associated with temporary overseas migration which started from the 1970s and became a huge source of revenue in the country since then (Ducanes, 2015). Globally, the main driver of temporary overseas migration is economic, specifically toward the improvement of the family’s standard of living and income insurance for the family (De Haas and Fokkema, 2010; Mesnard, 2000; Wong, 2006).

Previous studies acknowledge the impact of temporary overseas migration on the dynamics of transnational families with regards to parent–child relationship (Dreby, 2007; Wong, 2006), gender relations (Hoang and Yeoh, 2012; Hondagneu-Sotelo, 1992; Montes, 2013), and the provision of care among left-behind children (Asis et al., 2004; Bastia, 2009). Most of these studies examined transnational mothering (Arguillas et al., 2018; Garabiles, 2021; Madianou, 2012; Parreñas, 2005) focusing on the ways motherhood is redefined, challenged and maintained. However, non-migrant mothers often remained overlooked in studies of transnational families, despite their vital role in sustaining family life and providing care and emotional support to left-behind children (Galam, 2015; Gartaula et al., 2012; Nguyen et al., 2006). In this context, the present paper examines how non-migrant mothers from Filipino transnational families negotiate motherhood and authority in the absence of their migrant spouses.

## 2 Review of related literature

### 2.1 Filipino transnational families

Overseas Filipino Workers (OFWs) represent a significant demographic, with approximately 600,000 workers aged 45 and over comprising the largest share as of April to September 2023 (Philippine Statistics Authority, 2024). Their impact on the Philippines is substantial, with 12 percent of Filipino households having an OFW member (Mapa, 2020) and a total of $38.34 billion in remittances sent to families in 2024 (Fintech News Philippines, 2025). Recognizing their crucial economic contributions, former President Cory Aquino famously dubbed OFWs “Bagong Bayani” (modern-day Heroes) in 1988, a term that acknowledges their role in nation-building (Harvard International Review, 2023). This pervasive phenomenon gives rise to Filipino transnational families, defined as households where members reside in two different nation-states, typically with one parent working abroad while children and other family members remain in the Philippines (Parreñas, 2001).

Studies have increasingly explored how transnational families negotiate their long-distance relationships (see Evergeti and Ryan, 2011). Research consistently highlights the resilience of these families in maintaining cohesion across geographical distances. For instance, Cleofas et al. (2021) conducted a qualitative study showing how OFW families adapted their practices and interactions during the pandemic to reinvent and express their care for one another. Further illustrating this adaptability, Cabalquinto (2018) similarly conducted a qualitative study of Filipino workers in Australia, revealing how mobile devices significantly facilitate the formation and maintenance of intimacy among Filipino transnational families, enabling them to redefine their presence despite their absence.

Beyond general family adaptation, studies also delve into the challenges within these transnational families. Pinzon’s (2021) qualitative study on the left-behind children of OFW fathers illuminated the significant role of non-migrant mothers in mediating family relationships and sustaining communication within the transnational household. This focus on specific care practices is further elaborated by Gotehus (2023), who conducted a multi-sited qualitative study in Norway and the Philippines. Her research, which examined transnational caregiving and well-being among Filipino migrant nurses in Norway and their left-behind families, revealed the resilience of these nurses as they engage in a reciprocal of care with their aging parents in the Philippines, thereby strengthening their familial bonds.

However, despite these adaptive strategies, mother-away transnational families often face unique challenges, as the mother’s physical absence necessitates the practice of transnational mothering to provide care from a distance (Parreñas, 2001).

### 2.2 Mothering in transnational families

Research in the Philippines on transnational families primarily examines transnational mothers and their non-migrant husbands, revealing layered experiences within these family dynamics (Arguillas et al., 2018; Asis et al., 2004; Garabiles, 2021). Studies have delved into diverse aspects of family relationships, including gender negotiations between migrant mothers and their partners. Parreñas (2005) explored the gender paradox in Filipino transnational families, focusing on how the maintenance of mother-away transnational family structures challenges traditional gender roles assigned to women in domestic settings. Despite this challenge, caregiving practices within these family configurations perpetuate conventional notions of women’s domestic responsibilities. Additionally, external factors such as pressure from kin and community to conform to gender norms, cultural expectations to uphold societal standards of gender morality, and men’s resistance to expanding their roles in response to the formation of female-headed transnational families further complicate gender transformations within these contexts.

Madianou (2012) researched Filipina transnational mothers and discovered that information and communications technology (ICT) played a crucial role in enabling the practice of intensive mothering from afar and in managing the conflicting demands between work and family obligations. Additionally, the study highlighted that ICT usage served as a means to alleviate the heightened ambivalence experienced by these mothers, which stemmed from the cultural conflicts inherent in migration and motherhood. Similarly, in the study of Peng and Wong (2015), Filipina domestic workers in Hong Kong negotiate and maintain their motherhood roles despite geographical distances by relying heavily on telecommunication for coordinating with caregivers and husbands and engaging in intensive mothering as they adopt a hands-on approach. Baldassar et al. (2016) coined the term ambient co-presence to denote ICTs’ contribution to the maintenance of familial connections within transnational families that promote the sense of belongingness while at the same time carrying some implications on social surveillance.

### 2.3 Non-migrant wives

Previous studies on non-migrant wives looked at the impact of the husband’s migration on their wives’ role system (Chereni, 2015). Among Zimbabwean transnational families, wives of migrant workers negatively perceive their intensified parental involvement as the “new father,” considering that their role deviates from the gendered division of labor in the family after the migrant fathers’ return migration (Chereni, 2015). In South and Southeast Asia, the duties and responsibilities left by the male overseas migrant workers pose a burden among their wives as the latter have to fulfill tasks that traditionally their husbands perform (Asis et al., 2004). Moroccan wives of international migrant workers in nuclear households gained authority in decision-making but considered this expansion of roles to be a burden rather than emancipation (De Haas and van Rooij, 2010). Less studies is done to examine how non-migrant wives enforce and negotiate their authority on their adolescent children.

### 2.4 Mothering adolescent children

Existing research on motherhood has predominantly examined mothers’ ideologies and practices in raising young children, influenced by the prevailing intensive motherhood ideology (Barlow and Chapin, 2010; Guo, 2021; Jiao, 2019; Johnston and Swanson, 2006; McQuillan et al., 2008; Neyer and Bernardi, 2011; Ross, 2016; Valizadeh et al., 2018). This ideology considers motherhood as pivotal to women’s existence (Hays, 1996; Neyer and Bernardi, 2011). This essentialist discourse, influenced by patriarchy, emphasizes how motherhood is defined and controlled by men within a patriarchal society (Ross, 2016; Jiao, 2019). The concept of the “good mother” embodies these patriarchal definitions, shaping women’s identities, choices, and activities based on historical and cultural norms and expectations (Barlow and Cairns, 1997; Ross, 2016). Even when women resist, this ideology has a powerful institutionalized influence. Women face immense pressure to conform to these ideals and utilize them as benchmarks for evaluating their behaviors (Goodwin and Huppatz, 2010).

The ideals of motherhood often demand mothers to be child-centered and nurturing while contributing financially to their families. Hochschild and Machung (1989) highlight working mothers’ experience, which they referred to as the “two shifts,” encompassing paid employment and unpaid household and childcare tasks. This ascertains that women’s entry into the workforce does not free them from domestic responsibilities as long as traditional gender roles in households persist. McQuillan et al. (2008) found that mothers can value both work and motherhood simultaneously, with their evaluations of motherhood influenced by the constraints and challenges they face in different social spheres.

Despite the prevalence of studies on the ideology of good mothering, there is a lack of literature that explores the concept of motherhood across the lifespan, and the focus on mother-adolescent child relationships during adolescence, a critical period for identity formation and exploration, remains limited (Collins and Russell, 1991).

Mothering adolescent children is quite complex, given the incongruence in how mothers regard their mothering practice and how their adolescent children perceive them negatively as either too judgmental or too strict (Bojczyk et al., 2011). Di Stefano (2003) observed that mothers with adolescent children lose their omniscience, affecting their ability to monitor, influence, and control their children’s behavior. The difficulty of dealing with adolescent children was experienced by Iranian parents who ascertained that rebellion against parental authority and assertion of adolescent desire and authority result in conflict in the relationship (Valizadeh et al., 2018).

There needs to be more research specifically focused on the dynamics between non-migrant wives and their adolescent children in overseas Filipino families where the father is working overseas. Previous research on migrant-sending families has primarily investigated how migrant parents maintain and navigate their relationships with their children. Therefore, it is crucial to explore the complexities of the relationship between non-migrant wives in the absence of their OFW husbands.

## 3 Methods

This qualitative descriptive phenomenology study explores the narratives of mothering among left-behind members of Filipino transnational families, aiming to gather rich, detailed, and contextually grounded data that reveal the nuanced realities of parenting in the context of OFW families. This method allows informants to describe their experiences in their own words, providing a more authentic and subjective understanding of motherhood in the unique circumstances of OFW families.

The informants were selected using purposive sampling techniques with the following criteria: (1) mothers with adolescent children. The World Health Organization defines an adolescent as a child between 10 and 19 years old. Thus, mothers recruited have one child aged between 10 and 19. (2) They were legally married to an OFW. The study recruited legally married mothers to ensure the recognition of the work agency of the OFW husband for sending remittances to support the family. Overall, 20 mothers with adolescent children legally married to an OFW were interviewed as informants in this study.

Consistent with the narrative design, this study used an interview guide tool, the researcher uncovers the mother’s lived experiences and their perceptions of their condition, elucidates the significance of their experiences, and unveils their unique lived realities (Creswell and Poth, 2018). Interviews were conducted in Filipino and translated into English by the author.

The interview guide underwent a pre-test involving five mothers from non-OFW families, highlighting specific requirements. Although the feedback reveals that the questions adequately cover the intended topic, it showed a necessity for follow-up questions and a better understanding of the research instrument, mainly due to the challenge of non-linear flow in qualitative interviews. Questions were organized based on the research inquiries and categorized under distinct subheadings to address this. The instrument’s reliability was confirmed as informants consistently provided a similar understanding of the questions. This feedback highlighted the clarity and ease of answering the questions, indicating a consistent interpretation among informants regarding the intended inquiry.

The initial phase of analysis entails immersing oneself in the transcriptions (Cohen et al., 2000) which are repeatedly reviewed to familiarize oneself with the data and construct a narrative for each informant. Transcriptions are color-coded to streamline matrix preparation, with distinct colors denoting different thematic areas. After analyzing these matrices, codes are developed (Moustakas, 1994). Finally, themes are developed based on the different dimension of maternal authority. Simultaneously, the researcher sought related studies to compare and contrast the study’s findings with existing literature and ongoing debates. This process allowed for a comprehensive analysis and integration of the study’s results within the broader context of previous research.

This study employs reflexive thematic analysis, a method aligned with inductive analytical approaches that emphasize the exploration of theoretical assumptions influencing the analysis process (Braun and Clarke, 2021). Reflexive thematic analysis involves the identification of patterns within qualitative data, often manifesting as narratives derived from the diverse and nuanced perspectives of participants (Braun and Clarke, 2021).

The non-disclosure of personal characteristics are consistent with the research ethics of observing the privacy and confidentiality of information. Privacy is observed by withholding necessary information about the profile of the informant and their family to protect the reputation of the OFW family, as specific information about their financial situation, marital quality, child behavior, and quality of relationship with children may put the family at risk of embarrassment, distress, and shame. To ensure that the relationship with children is not affected by the informants’ self-report of child behavior and the quality of their relationship, the researcher will prevent children’s access to the personal information and accounts shared by their parents. All information remain private and for academic purposes. If informants decide to cancel or withdraw from participating in the study for personal reasons, they will be allowed to do so. Those who participated in the study signed the informed consent form to confirm their awareness of the nature of the study.

One methodological limitation of this study is associated with positionality, which refers to the dynamic ways individuals are defined by socially significant identity dimensions (Secules et al., 2021). It is important to note that all informants are spouses of OFWs specifically located in the province of Nueva Ecija. The researcher’s positionality as a resident and college teacher from the locale being studied, as well as being a mother of an adolescent child, inevitably shapes the outcome of the study on mother-adolescent child relationships. Therefore, the researcher simultaneously embodies an outsider and insider perspective in relation to the study. The etic perspective, emphasizing detached observations, arises from the fact that the researcher does not belong to a transnational family. Conversely, the emic perspective is illustrated by the researcher’s familiarity with the local context, providing a nuanced understanding of socio-cultural dynamics and familial norms.

This insider perspective enables navigation of the research terrain with sensitivity and insight, potentially facilitating deeper connections and richer data collection. Additionally, the researcher’s dual role as a college teacher and a mother offers a unique perspective on parent–child interactions, informed by both academic knowledge and personal experience. However, it is imperative for the researcher to critically reflect on how her positionality may influence her interpretations and biases, striving for reflexivity and ensuring that her insider status does not inadvertently sway the objectivity of the study’s findings.

Mayring (2007) posits that phenomena are inherently bound by time and context. As a result, any analytical generalizations derived from the findings can yield moderatum generalization (Mayring, 2007). This underscores the importance of and interpretation in extrapolating generalizations from qualitative data. Consequently, the findings may not conclusively apply to the same population in a different context or to other populations of non-migrant mothers across diverse geographical areas.

While remote qualitative data collection, such as conducting online interviews during the pandemic, is both relevant and practical, it comes with its drawbacks. Datoon et al. (2022) highlight the challenge of building rapport with respondents during phone interviews. Unlike face-to-face interviews, which may foster rapport and encourage informants to share more of their lived experiences, remote methods may not facilitate the same level of connection.

A more deliberate and focused descriptive-qualitative study specifically examining the mother-adolescent daughter or mother-adolescent son relationship could have provided greater insight into the lived experiences of non-migrant wives. Additionally, including younger children in the study might have added nuance to the findings.

## 4 Results and discussion

### 4.1 Discourses of authority: a cultural analysis of parental influence over adolescents in Filipino transnational families

Traditionally, fathers are seen as the primary disciplinarians in Filipino families. However, in their physical absence, the dynamic of parental authority become more complex, particularly during adolescence—a period marked by intense negotiation of parental control and children’s autonomy. Consequently, the task of raising adolescent children in Filipino father-away transnational families demands the enforcement of maternal authority, largely due to deeply embedded cultural norms.

Authoritarian parenting has always been a cornerstone of child-rearing practices in Filipino families, and disciplinary strategies are profoundly shaped by the cultural emphasis on parental authority and filial piety, with children expected to demonstrate obedience as a sign of respect (Tarroja, 2010). This cultural expectation directly impacts non-migrant mothers, who are anticipated to raise well-mannered, obedient, God-fearing, and respectful children to be recognized as “good mothers.” This aligns with familism among Filipinos, which mandates that non-migrant mothers and their left-behind children cultivate strong family cohesion. This is further underpinned by the collectivist nature of Filipino society, where interpersonal relationships and family ties are highly valued (Yau and Smetana, 2003).

Within this collectivist context, the interplay between individual autonomy and parental authority significantly influences adolescent development. Filipino adolescents often exhibit a strong sense of deference to parents, and internalize strong family hierarchy. This predisposition to respect parental authority and downplay their autonomy, as observed by Fuligni (1998), significantly contributes to family cohesion and mitigates family conflict (Fuligni, 1998).

Non-migrant mothers in Filipino transnational families face a complex dilemma: they are caught between enforcing maternal authority to raise well-behaved children and be considered “good mothers,” while simultaneously striving to maintain a positive relationship with their adolescent children, who, in turn also consider them “good mothers.” Thus, these mothers frequently straddle the line between disciplining their adolescent children and granting them autonomy and room for negotiation. This inherent tension explains why non-migrant mothers employ various strategies to enforce their authority. These include adopting authoritarian parenting styles, leveraging the authority of their OFW husbands, and even utilizing approaches that often involve negotiating with their adolescent children for compliance by routinizing their daily schedules and allowing adolescents to negotiate.

#### 4.1.1 Authoritarian parenting style in Filipino transnational families

The physical absence of the OFW husband necessitates that mothers assert their authority over their children, often employing authoritarian mothering techniques characterized by the implementation of strict rules, reprimands, persistent nagging, and other disciplinary actions (Baumrind, 1991; Darling and Steinberg, 1993; Monaghan et al., 2012). According to Kuhar and Reiter (2013), the parental control paradigm frequently includes behavioral control strategies such as exerting parental pressure and domination over their children. Within this framework, children’s behavior is frequently interpreted as an indicator of mother’s success in child rearing. Consequently, to cultivate obedience and instill good moral values, non-migrant mothers tend to adopt an authoritarian parenting style. Mothers themselves acknowledge that adherence to their directives is more readily achieved through the implementation of stringent parenting approaches.

Mothers in this study demonstrate a firm hand in parenting, emphasizing strict adherence to rules and demanding unconditional compliance from their adolescent children. Mothers of early adolescents face the challenge of navigating strategies to elicit compliance from their children. Some resorted to stern gazes, verbal reprimands and persistent nagging. One of the informants revealed that, “*when I give orders, I have to raise my voice for them to follow me. I have to get angry*.” Consistently, these mothers observe that they should be strict in giving instructions. If they wanted their children to follow them immediately, they have to nag them. Cherry shares that she employs diverse methods to assert her authority and secure obedience from her 12-and 13-year-old sons. According to her, “*I give them a stern look while raising my voice, adding a slight threat. Nagging proves effective*.”

Authoritarian mothering also entail gender-differentiated restrictions, particularly for daughters, such as prohibitions against entertaining suitors and limitations on time spent on chatting and social media. To ensure compliance, mothers implement minor disciplinary actions, exemplified by the confiscation of a daughter’s phone for staying out late. This protective approach toward girls, aimed at ensuring their “safety and welfare,” aligns with broader cultural norms identified by Liwag et al. (1998).

However, a key finding of the present is that the effectiveness of these maternal control strategies is significantly associated with the non-migrant mother’s financial contribution to the family. Specifically, the mother’s proportionate contribution relative to her husband’s income appear to enhance her perceived authority over their children. This aligns with established sociological literature demonstrating that a women’s increased contribution to household income is linked to both greater personal autonomy and enhanced household control, with her bargaining position strengthening considerably when her earning surpass her husband’s (Alcantara, 1994).

Consider Khaye, whose husband, employed at an Aluminum company in Saudi Arabia, typically remits a modest $200 to $250 monthly. For 11 years, Khaye has worked as a house helper to supplement this income. Through her diligent savings from her husband’s remittances and her own earnings, she has been able to fund significant home repairs and acquire motorcycles for her children. She proudly states that, “*I have built up our home with my husband’s small remittances. My rationale then was, even if he sent little, someday he will come home. He probably thought we were living comfortably. Just look at my house now – it was empty before, but now it is complete, and we even have motorcycles. One is already fully paid for, and I am still paying for the other one*” (translated by author).

Khaye’s substantial financial contributions have significantly solidified her authority within the family. With this, she informed her children not to have vices. She said, “*They also do not have any vices, and I told them that if they ever develop one, they should support themselves – but if they will just rely on me, that is what I do not want*” (translated by author).

Within the authoritarian parenting framework, mothers are expected to act as disciplinarians, clearly delineating boundaries between acceptable and unacceptable behavior. Lety, a midwife in her town, believes mother should win over their adolescent children. But she stressed that despite the need to understand their adolescent children’s interests, mothers must still be strict and firmly impart that incorrect actions are unacceptable.

Some mothers wanted their children to adapt a routine where volunteering for household chores is part of. Filipino non-migrant mothers such as Uning promotes efficient routine among her children. The statement below highlights mothers’ expectation for children to contribute in managing their dishes and other household chores. She shares her frustration:

“I really prefer things done in the morning. When they wake up, I want them to eat right away. That way, I have already finished cooking and laundry. But then after they eat, they just dump their plates in the sink, that is what makes me upset. You know how kids are – they do not like being reprimanded. When they hear me raising my voice and getting mad, they do not like that and eventually the dishes get washed” (translated by author).

Tina stood out among the non-migrant mothers for her direct approach in correcting both her children and their adolescent friends, particularly concerning behavior she deemed inappropriate. She articulated her perspective: *“Such behaviors were prevalent when they were at school, where group activities were common among boys and girls. One time they stayed here to do their assigned group task. My concern was when the boys acted out flirtatious or improper. My preference was simply for them to be well-behaved”* (translated by author).

Filipino non-migrant mothers sometimes resort to physical discipline when they lose their temper, aiming to gain their children’s compliance. This loss of temper in dealing with children is associated with the marital relations quality between OFW fathers and non-migrant mothers. One mother, who just recently severed her relationship with her OFW husband due to his infidelity, recounted a past incident where she struggled to control her anger. She recalled pulling her adolescent daughter’s hair, emphasizing the intensity of her rage, illustrating spillover effect of marital separation to the parenting style of non-migrant mothers.

#### 4.1.2 Leveraging the authority of OFW fathers

In the context of Filipino transnational families, geographical distance of OFW fathers does not necessarily diminish patriarchal authority, instead this study suggests that their remote influence significantly reinforces the disciplinary power of non-migrant mothers. This collaborative disciplinary approach is illustrated by Myra, a participant, who explained, “*They are monitored by their father. I inform him about their behavior when they are stubborn. Consequently, both of us (referring to herself and the father, implied) engage in scolding them*.” This remote reinforcement of parental authority proves particularly functional in contexts where non-migrant mothers face challenges in establishing or asserting disciplinary control, particularly over adolescent sons. The father’s perceived oversight provides an external locus of control that augments the mother’s immediate authority. When adolescent children seek permission for activities like sleepovers or outings, mothers frequently deny these requests. Should children persist, mothers commonly invoke the father’s disapproval as the definitive reason for refusal. This strategy, “*their father did not permit it*,” effectively curtails further negotiation. In this sense, the distant father’s decision becomes a crucial tool for maternal boundary setting and reinforces compliance. Thus, by actively involving the OFW father in daily monitoring and disciplinary feedback, non-migrant mothers are empowered through the implicit validation and backing of the father’s overarching authority. This positions non-migrant mothers not merely as surrogate caregivers but as direct extensions of the father’s disciplinary presence, leading greater weight to their directives. This practice does not only reinforce the traditional role of Filipino fathers as strong disciplinarians but also crucially maintains their influence across borders, thereby bolstering the mother’s direct authority within the household in their physical absence.

#### 4.1.3 Chore delegation and adolescent engagement in low-income Filipino transnational families

In low-income Filipino transnational families where fathers are absent, hiring house helpers is uncommon due to financial constraints on remittances. Consequently, non-migrant mothers bear the primary responsibility for household chores. To ease this load, mothers often pass on maternal obligations to their adolescent children. It is particularly common for working Filipino mothers to delegate substantial household chores, to their eldest daughters, especially in the absence of paid domestic help and support from female relatives. Lety, a midwife, illustrates this dynamic: “*I trained my eldest daughter early to help me with household chores. That is where I find relief. When I am not around, she is the* ‘*little mother*’ *in our house. She cooks, washes clothes, and takes care of her younger siblings*” (translated by author).

Recognizing that *“kids can be a bit lazy about doing their chores”* (translated by author) some mothers create daily task schedules to involve all their adolescent children. Given these circumstances, Filipino non-migrant mothers often strategize to ensure their children’s engagement in household chores. This is particularly crucial for equitably distributing tasks, moving beyond the common reliance on eldest daughters for domestic work. Illustrating this, one mother recounted how establishing a chore timetable empowered her to instruct her son to cook rice and wash dishes at lunchtime. She elaborated, “Whatever tasks is assigned to them, they do it*. My son is in charge of cooking rice. When lunchtime comes, he clears the plates”* (translated by author).

The involvement of adolescent children in Filipino transnational families in household chores can sometimes be met with subtle resistance, evident in delays or task neglect. Consequently, non-migrant mothers may adopt a stricter, more authoritarian parenting approach.

#### 4.1.4 Negotiating authority: maternal strategies for influence and compliance in Filipino transnational families

Since the perceived legitimacy of maternal authority decreases with late adolescents, mothers negotiate with their children instead (Bernardo, 2010). Filipino non-migrant mothers leverage negotiation as a key tool for enforcing maternal authority, primarily through persuasion and appeals for family. This approach helps them prioritize their adolescents’ education over romantic relationships and secure their active participation in household chores. Lisa, who experienced early teenage pregnancy shares her negotiation with her daughter: *“We have an agreement: if you have a boyfriend, you will not study, and if you study you will not have a boyfriend*” (translated by author). This negotiation reflects her proactive strategy of addressing the potential disadvantages associated with early commitment. Although options are given to their daughters, this strategy reflects how they negotiate and influence the choices of their adolescent children. Bernardo (2010) explains that in Filipino culture, parents’ expectations and control over their children’s educational striving are seen as normative and legitimate. Thus, parental goals often drive Filipino students’ achievement motivations.

In the sphere of household management, negotiations often manifest as appeals, particularly among non-migrant mothers who frequently eschew physical punishment in favor of more discursive strategies. Instead, these mothers engage in persuasive appeals and collaborative negotiations to secure their adolescents’ participation in housework. This approach highlights a reliance on relational dynamics rather than overt coercion. For instance, one mother’s appeal to her early adolescent son vividly illustrates this: “*We need to help each other, and it is just us. No one else will help us because we do not have anyone else here. We do not have a helper. So, we really need to help each other”* (translated by author). This appeal not only underscores a sense of shared responsibility but also subtly invokes the absence of external support, framing housework as a collective endeavor essential for the family’s functioning. Such interactions are crucial for the social reproduction of domestic labor, where contributions are solicited through appeals to familial solidarity and mutual aid.

Furthermore, mothers often frame these negotiations as parental advice, subtly shifting the dynamics from a directive guidance to the child’s own benefits. Consider Tina, whose children wear eyeglasses at a young age. She advises them to go to bed early, acknowledging their late nights due to gadget use, and explains that her guidance is ultimately for their own well-being. This strategy positions the mother’s request within a framework of nurturing care and foresight, encouraging compliance through an appeal to child’s self-interest rather than simple command. This demonstrates how mothers utilize affective strategies to navigate household responsibilities and promote desired behaviors without resorting to punitive measures.

#### 4.1.5 Navigating maternal authority: negotiation and autonomy in Filipino transnational families

The willingness to challenge maternal authority is shaped by the family environment and the cultural norms of parenting (De Leon Born and Vasbø, 2025). Although Filipino non-migrant mothers adopt authoritarian parenting strategy, some of them allow their children to negotiate with them. Negotiations can be a form of young people’s resistance to parental control (Solomon et al., 2002). Children are capable of negotiating with their parents. For example, Tina’s children expressed a preference to spend their vacation bonding only with their immediate family, rather than with their cousins, because they did not want their achievements to be compared. Children can also negotiate their responsibilities at home, especially when they are busy with schoolwork. Pinky has noticed a pattern: her daughter gets frustrated and retreats to her room, saying “You always give orders Mama,” whenever Pinky issues too many commands. This behavior highlights a common dynamic with adolescent children, who often resist an authoritarian parenting style. Pinky’s daughter is clearly pushing back against her mother’s perceived controlling approach.

Negotiation between Filipino adolescents and their mothers illustrates how personal autonomy can co-exist with legitimate maternal authority within Filipino transnational families. However, this dynamic is primarily observed among late-adolescent firstborns. Bernardo (2010) explains that such negotiations occur with late adolescents due to weaker perception of parental authority among older adolescents, allowing them to negotiate the domains their mothers can regulate.

## 5 Conclusion

In Filipino transnational families, non-migrant mothers face a complex challenge: they must embody the “good mother” ideal while navigating the physical absence of their husbands. This ideal is deeply rooted in a culture where authoritarian parenting is a cornerstone of child-rearing. Children are expected to show filial piety and obedience as a sign of deference to parents, and their demeanor is often seen as a direct reflection of their mother’s success. To be considered “good mothers,” non-migrant mothers often assert their authority through authoritarian parenting styles. This can involve common disciplinary tactics like nagging, reprimanding, and stern gazes, all aimed at raising well-mannered children. The study found that a mother’s authority is significantly bolstered by her financial contributions to the family, even when her husband sends remittances. Additionally, mothers effectively leverage the remote influence of the OFW husband. They used his perceived oversight as an external source of control, reinforcing their own directives and ensuring compliance from their children. However, the “good mother” in the Filipino context also involves cultivating a positive relationship with her children. As such, non-migrant mothers increasingly employ negotiation strategies. They appeal to their children’s sense of family solidarity and welfare to encourage contribution to housework, while also allowing adolescents the space to negotiate and exercise their own growing autonomy. This delicate balance highlights the evolving nature of maternal authority in these unique family structures.

This study contributed to family research through a comprehensive understanding of the interplay between culture, migration, family dynamics, and adolescent development that ultimately informs more effective support system for transnational families. The findings regarding mother’s financial contribution and their enhanced authority demonstrated how economic empowerment increased into maternal influence. Future studies may examine the long-term effects of the mediated authority on adolescent development and family cohesion as well as how negotiations in mother-adolescent child relationship evolves over time in transnational families.

## Data Availability

The raw data supporting the conclusions of this article will be made available by the authors, without undue reservation.
